# Automatic identification of early ischemic lesions on non-contrast CT with deep learning approach

**DOI:** 10.1038/s41598-022-22939-x

**Published:** 2022-10-27

**Authors:** Prasan Kumar Sahoo, Sulagna Mohapatra, Ching-Yi Wu, Kuo-Lun Huang, Ting-Yu Chang, Tsong-Hai Lee

**Affiliations:** 1 Department of Computer Science and Information Engineering, Chang Gung University, Taoyuan, Taiwan; 2 Department of Occupational Therapy, Graduate Institute of Behavioral Sciences, College of Medicine, Chang Gung University, Taoyuan, Taiwan; 3 Department of Neurology, Chang Gung Memorial Hospital, Linkou Medical Center, No. 5, Fu-Hsing Street, Guishan, Taoyuan, 333 Taiwan; 4 College of Medicine, Chang Gung University, Taoyuan, Taiwan

**Keywords:** Biotechnology, Computational biology and bioinformatics

## Abstract

Early ischemic lesion on non-contrast computed tomogram (NCCT) in acute stroke can be subtle and need confirmation with magnetic resonance (MR) image for treatment decision-making. We retrospectively included the NCCT slices of 129 normal subjects and 546 ischemic stroke patients (onset < 12 h) with corresponding MR slices as reference standard from a prospective registry of Chang Gung Research Databank. In model selection, NCCT slices were preprocessed and fed into five different pre-trained convolutional neural network (CNN) models including Visual Geometry Group 16 (VGG16), Residual Networks 50, Inception-ResNet-v2, Inception-v3, and Inception-v4. In model derivation, the customized-VGG16 model could achieve an accuracy of 0.83, sensitivity 0.85, F-score 0.80, specificity 0.82, and AP 0.82 after using a tenfold cross-validation method, outperforming the pre-trained VGG16 model. In model evaluation, the customized-VGG16 model could correctly identify 53 in 58 subjects (91.37%) including 29 ischemic stroke patients and 24 normal subjects and reached the sensitivity of 86.95% in identifying ischemic NCCT slices (200/230), irrespective of supratentorial or infratentorial lesions. The customized-VGG16 CNN model can successfully identify the presence of early ischemic lesions on NCCT slices using the concept of automatic feature learning. Further study will be proceeded to detect the location of ischemic lesion.

## Introduction

Prompt identification of ischemic lesion is crucial for triaging patients as potential candidates for thrombolysis due to the narrow therapeutic time window. Early therapeutic intervention may improve stroke outcomes and lower the risk of recurrent stroke by as much as 80%^1^. Urgent brain imaging study is suggested to be performed on first hospital arrival in patients with suspected acute stroke^2^. Non-contrast computed tomogram (NCCT) is the most commonly available tool in emergency department for the initial assessment and can exclude intracerebral hemorrhage from thrombolysis therapy. However, NCCT has the limitations to identify early signs of cerebral infarction and evaluate the ischemic lesion in infratentorial region^3^. Brain Magnetic resonance (MR) imaging is suggested a good imaging tool for the early identification of ischemic lesion^4,5^. However, MR imaging is limited due to low availability, high costs, and long acquisition time^5^.

The development of automatic method using the concept of artificial intelligence (AI) to efficiently differentiate the brain lesions has been investigated in recent years. Deep learning (DL), a subset of machine learning, possesses the ability to learn abstract, high-order features from data without requiring feature selection independently through neural networks^6,7^. The DL using convolutional neural network (CNN) is suggested being good at image classification in medical imaging^8^ and can automatically identify patterns in complex imaging datasets without the need for direct human interaction during training process^7^.

The present study intends to develop an automatic model to detect the presence or absence of ischemic lesion on NCCT slices to identify the ischemic stroke patients by using the concept of DL-based CNN network.

## Methods

### Study population

The NCCT and corresponding MR images of ischemic stroke patients and normal subjects were retrospective collected from a prospective image registry of Chang Gung Research Databank during the period of 2014 to 2019 at Linkou Chang Gung Memorial Hospital, Taiwan. The inclusion criteria were (1) ischemic stroke patients with no visible infarction on first-line NCCT which was done within 12 h after stroke onset and positive DWI/ADC lesion on subsequent brain MR image^9^ reported by the neuroradiologist, (2) normal subjects with negative infarction lesion on NCCT and negative DWI/ADC lesion on brain MR image reported by the neuroradiologist; (3) the interval between NCCT and MR imaging was within two weeks (mean ± SD = 7.4 ± 5.3 days); (4) during this 2-week period, there was no new brain event; (5) no motion artifact in NCCT and MR/DWI images. The exclusion criteria were (1) infarction size on MR/DWI < 0.5 cm and (2) patients with traumatic brain injury, brain malignancy, intracerebral hemorrhage and vascular anomaly. In the collection of brain NCCT, first, we checked the regular radiology reports which concluded no visible infarction by neuroradiologist. Second, the brain NCCT was re-confirmed by two neurologists who also agreed there was no visible infarction after the assessment of inclusion and exclusion criteria. Third, the eligible brain NCCTs were collected and de-identified before deep learning approach. The reports by neuroradiologists were regarded as the gold standard for both inclusion and exclusion criteria. In case, there was conflict among neuroradiologist and neurologists, the images were not included for analysis (the inter-observer difference near 100%). Our institution review board (IRB) of Linkou Chang Gung Memorial Hospital approved this study (IRB No. 201900028B0, 201900048B0). Informed consent was obtained from all subjects. All methods were carried out in accordance with relevant guidelines and regulations.

### Image acquisition

Digital Imaging and Communications in Medicine (DICOM) images were used. Each image is 512 × 512 pixels in size. The original NCCT hounsfield unit (HU) was transformed with a brain/sinus window (center 40HU, width 150HU) into 256 Gy levels. NCCT was performed on detector CT scanner (Aquilion 64, Toshiba, Japan) with slice thickness 5 mm. MR image was performed at 3.0 T scanner (Ingenia 3.0 T MR system, Philips, USA).

### NCCT image preprocessing

**Figure 1:**
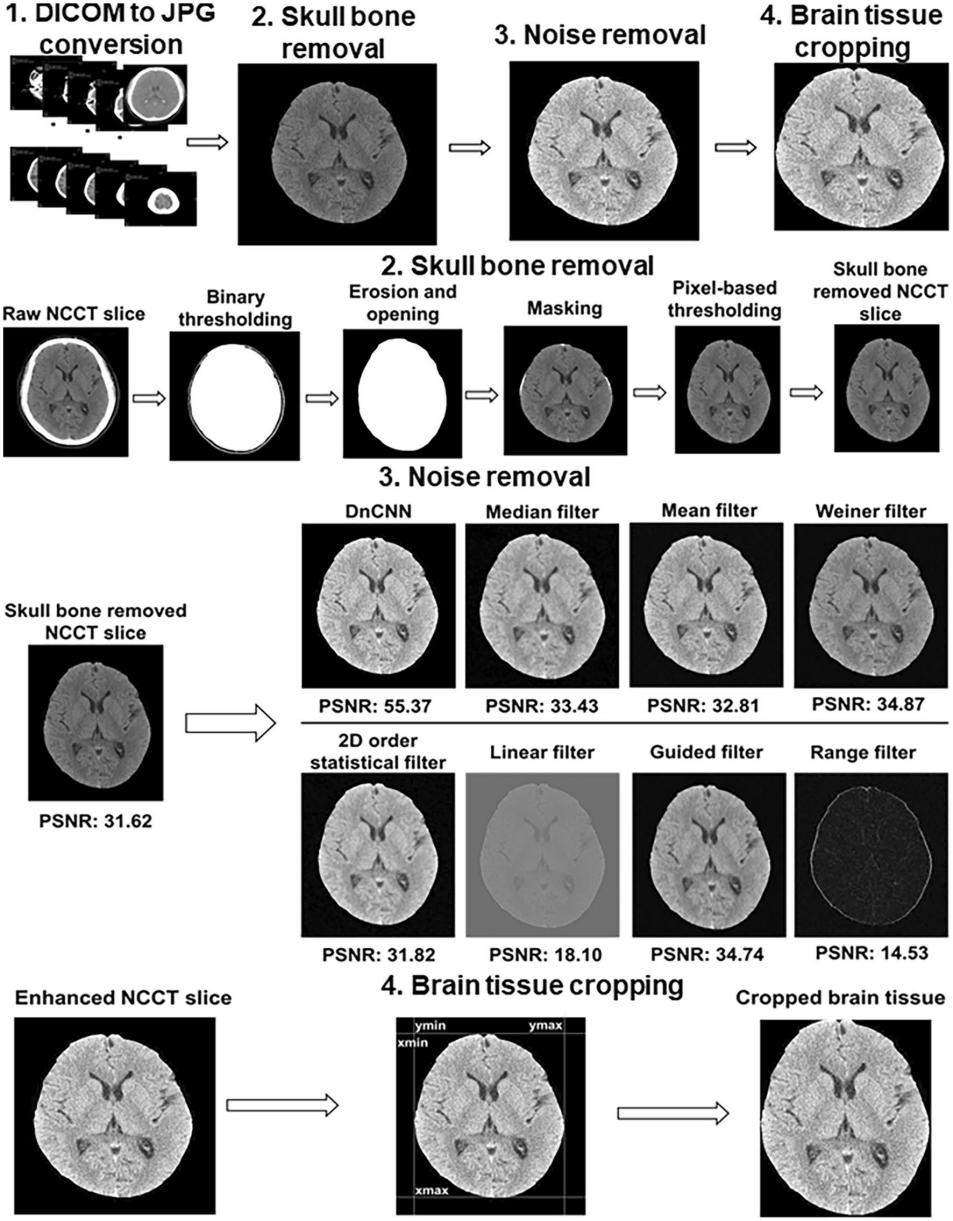
Procedures of NCCT image preprocessing. The upper part shows overall preprocessing steps starting from 2D NCCT slices construction to brain tissue cropping. The next part conveys the intermediate-steps used for skull-bone removal. A comparison of PSNR for different noise removal algorithms are presented in the 3rd part. The final part displays the selection of rectangular non-brain tissue region for cropping of the exact brain tissue part. *NCCT* non-contrast computed tomogram, *2D* two dimensions, *PSNR* peak-to-signal noise ratio.

The identification of the presence of ischemic lesion on raw NCCT which was taken in the early stage after ischemic stroke using DL is challenging due to low contrast quality, presence of skull bone, and in-built noise artifacts. Therefore, the following preprocessing steps were used (Fig. 1) before DL model design to improve the NCCT image quality and remove unwanted information.

### Construction of 2D NCCT slices from DICOM to Joint Photographic Expert Group (JPEG)

During the period of ischemic stroke within 12 h after onset, the early ischemic sign could be subtle, and the ischemic density could be similar to the surrounding brain tissue. Besides, the extraction of voxel from the lacunar infarction is challenging, and the training process of 3D slices is more resource-exhaustive than 2D. This study selected deeper 2D pixel-level analysis instead of 3D voxel-level by converting the DICOM images to JPEG images using the software RadiAnt DICOM Viewer (https://www.radiantviewer.com/). The 2D JPEG images were used for analysis as JPEG compression format is good for faster processing and is widely accepted for medical image analysis without compromising the image quality. During the conversion, the standard 8-bit grayscale depth (0–255) was maintained with the source pixel dimension of 512 × 512. In addition, each converted slice was verified by experienced neuroradiologist and neurologist to ensure there was no eye-catching distortion of the brain tissue.

### Ischemic NCCT slice selection

In the case of early ischemic stroke with an onset time < 12 h, it is challenging to identify the ischemic lesions on the first-line NCCT slices. Besides, the collected DICOM NCCT data consisted of 25–40 slices per examination, and the ischemic lesions might not appear in all the slices. Therefore, the ischemic NCCT slice selected for model selection, derivation and evaluation was carried out by using the respective MR-DWI sequence as the reference images. The mapping between NCCT and DWI image modalities was performed in consideration of various cerebral features including the appearance of the ventricle, sulcus, brain structure, mid-line, and order of the imaging sequences (Supplementary Fig. S1). In some conditions when the exactly mapped NCCT slices to DWI were not available, the nearest matched slices were considered to identify the ischemic lesion.

### Removal of skull bone

Bony skull and falx calcification were removed with the preservation of brain tissue. First, binary thresholding was used over raw NCCT to get the outer part of skull. Second, morphological operations including erosion and opening were performed to remove falx calcification and outer brain region (mainly skull). Third, masking operation was carried out to obtain the exact brain tissue. Finally, skull bone was completely removed after applying the pixel-based thresholding. The automatic algorithm of skull bone removal was designed by using MATLAB R2019a (https://www.mathworks.com/products/new_products/release2019a.html).

### Noise removal and image enhancement

In the processing of digital images, the amount of internal in-built noise is always unknown. Therefore, the amount of known noise needs to be added before noise removal. In the case of NCCT slices, the Gaussian noise is mostly preferred for addition^10^. The added noise affects the internal pixel characteristics of the original image, especially the mean and variance factors. Hence, we added the Gaussian noise with the default value of mean = 0 and variance = 0.001. The amount of noise was quantified using the concept of Peak-to-Signal Noise Ratio (PSNR)^11^. Mathematically, the PSNR value is inversely proportional to the amount of noise. The higher the PSNR value; the less the presence of noise in the image, implying the image is more enhanced and noise-free. Initially, in the processing of NCCT slices, the noise quantification using PSNR value was measured by passing the NCCT slices before and after the noise addition. In this instance, the original NCCT slices with unidentified noise obtained after skull removal acted as a reference for the known-noise added NCCT slices. To select the appropriate noise-removal method, different conventional filtering algorithms as well as denoising convolutional neural network (DnCNN) (https://www.mathworks.com/help/images/ref/dncnnlayers.html) developed by using MATLAB R2019a were compared. The quality of the noise removal was verified by comparing the measured PSNR value of the denoised slice with the original noisy slice. The original noisy slice was used as a comparable parameter to determine the improvement in the enhancement. In the present study, we used the DnCNN algorithm which could perform better with PSNR = 55.37, making the image more enhanced compared to the input NCCT slice with only skull bone removal (PSNR = 31.62).

### Brain tissue cropping

To eliminate the background surrounding brain tissue, automatic cropping of brain tissue was performed by using the concept of pixel-level analysis (https://www.mathworks.com/matlabcentral/answers/397432-auto-crop-the-image). The cropping was performed as a rectangular area where the coordinates Ymin and Ymax represent the lowest and highest nonzero columns, whereas the Xmin and Xmax indicate the minimum and maximum nonzero rows in an image size of 512 × 512. In the final phase, a rectangular cropped brain tissue region was obtained using the coordinates (Xmin, Ymin): (Xmin, Ymax), (Xmax, Ymin): (Xmax, Ymax) as shown in Fig. 1.

### Development of DL-based automatic identification algorithm

The CNN model for identifying ischemic and normal slices was designed based on the concept of supervised learning^7^ to train the machine, and both ischemic and normal labels were given to the pretrained CNN for learning purpose. This pretrained CNN model has already been trained with a large ImageNet dataset (https://www.tensorflow.org/) and is reusable for the analysis to obtain faster and reliable results.

### Implementation of environment

The implementations were carried out using the GPU version of TensorFlow 1.14 with the specification TITAN RTX 24 GB × 4, Intel^®^Xeon^®^Scalable Processors, 3UPI up to 10.4GT/s, with 256 GB memory, Nvidia-smi 430.40 in Ubuntu 18.04.3 platform. In addition, various predefined libraries such as Keras = 2:1:6, python = 3:6:9, numpy = 1:18:4, matplotlib = 3:2:1, OpenCV = 4:1, pillow = 7:1:2, and scikit-learn = 0:21:3 were used during image analysis.

### Establishment of CNN-based identification model

The establishment consisted of three phases including model selection, derivation and evaluation. Among the entire 675 considered subjects, patient-wise data splitting was performed where 617 subjects including 517 ischemic stroke patients and 100 normal subjects were considered for model selection and derivation. For the rest 58 subjects, balanced data of 29 ischemic stroke patients and 29 normal subjects were chosen randomly for model evaluation.

For model selection and derivation of appropriate CNN model, 1631 ischemic NCCT slices from 517 ischemic stroke patients and 1808 normal NCCT slices from 100 normal subjects were collected after confirming with the corresponding MR images. Another 476 NCCT slices showing no evidence of DWI/ADC lesions on corresponding MR images were also acquired from 517 ischemic stroke patients, resulting in a total of 2284 normal NCCT slices.

### Model selection

To select appropriate CNN model, slice-wise data splitting was performed before building the model. One-fold of the data in the ratio of 90:10 among total slices was selected randomly for model selection. Five CNN models were evaluated including Visual Geometry Group 16 (VGG16), Inception-v3, Inception-v4, Residual Networks 50 (ResNet 50), InceptionResNet-v2 (IR-v2), which were already trained in large ImageNet dataset and customized using transfer learning^12^.

### Model derivation with selected VGG16 model customization

To increase the performance of the selected pretrained VGG16, appropriate customization was performed including the addition of two batch normalization layers after each dense layer, which were not used in the standard VGG16 model. The batch normalization layer implements a transformation that normalizes the mean output value close to 0 and the standard deviation close to 1 by subtracting the mean and dividing the standard deviation for every mini-batch obtained from the previous output layer^13^. This study used batch normalization layer to accelerate the training process, provide the regularization effect and reduce the estimated error. In addition, the standard Softmax activation function used in the last output layer was modified to Sigmoid which is most preferred for binary classification^14^ and might be suitable for the feature differentiation between our ischemic and normal NCCT slices. To further optimize the model performance, Adam optimizer was used to help the computation of learning rates for each parameter adaptively with lower requirements of hardware and computational resources^13^. In addition to minimize the loss and increase the performance, categorical crossentropy loss function was employed when the inputs were encoded in the form of one-hot vector like $$\left[\begin{array}{c}1\\ 0\end{array}\right]$$ and $$\left[\begin{array}{c}0\\ 1\end{array}\right]$$ for ischemic and normal slices, respectively^15^.

### Hyper-parameter tuning

The random search technique was applied for hyper-parameter tuning as it outperforms the traditional grid search technique^16^. Ultimately, a fine-tuned model was obtained by setting the optimal values of hyperparameters such as learning rate = 0.001, batch size = 8, number of epochs = 4, number of steps per epoch = 1000, dropout rate = 0.5, decay = 0.01, epsilon = 0.0000001, momentum = 0.9 and the number of neurons in the dense layer = 1049.

### Adopted data augmentation methods

Usually, the ischemic pattern may vary with the location, territory, size of infarction, and onset time. In the case of early ischemic stroke, the ischemic injuries are frequently invisible and could be similar to the surrounding normal brain tissue. To understand the NCCT image in different scales, directions, angles and magnification, CNN model was used through the concept of data augmentation^17^. In the customized-VGG16 model, the data augmentation through rescaling of the value = 1/255 was applied to normalize the input pixel from the range [0, 255] to [0, 1]. This rescaling factor enabled the NCCT slices to contribute more evenly to the total loss. This rescaling method also helped the model for faster processing of the data.

The channel-shifting parameter with the value = 8 was adopted to change the default color channel of the image. Based on this shifting value, a certain amount of color was added to the slice to make it more prominent. This augmentation effect enabled the machine to judge any deviation in the color pattern for both ischemic and normal regions. The horizontal-flip = True augmentation parameter was used to compare the minute changes in contralateral side of the hemisphere. Further, there might be motion artifact during NCCT acquisition, and a slight rotation with 3 degrees was applied to get the knowledge of ischemic injury from a different view. In the case of small infarction such as lacune especially in the corona radiata and basal ganglia regions, a zooming operation with zoom-in value = 1.0 and zoom-out value = 0.7 was employed. The values of different augmentation methods were decided by performing several rounds of experiments considering multi-parameterized NCCT data.

### Adopted cross-validation strategy

To develop an unbiased generalized CNN model with appropriate hyperparameter tuning, the concept of k-fold (k = 10) cross-validation was used with k-rounds of training and testing. In each iteration, one data fold (Fold 1) was assigned to the testing set, and the remaining 9 folds (k − 1) were used as the training set^18^ (Supplementary Fig. S2). Further, 10% of data from the whole training set was randomly assigned to the validation set in each iteration to introduce the concept of early stopping and proper hyperparameter tuning. In the present study, the validation set was considered for model tuning, and the testing set was used for model evaluation in the individual round. To derive the best model, different combinations of hyper-parameters were investigated and evaluated via tenfold cross-validation. The customized-VGG16 model was trained by considering each random combination of tuned hyperparameters, where after the completion of 10 iterations, the performance was evaluated by considering the average value of total 10 folds for each evaluation metric. This process of hyperparameter tuning for performance improvement was performed iteratively until a competent outcome was achieved. After comparing the tenfold average value of the performance metrics for different combination of hyperparameters, the best model with the smallest validation loss and the highest average value of performance metrics was selected and served as the final model for the patient-wise evaluation in the next phase (Supplementary Fig. S2).

### Model evaluation

To evaluate the derived model, the available NCCT slices of individual patient were given as input. Upon identifying any NCCT slices, the corresponding subject was recognized as ischemic, otherwise normal. For model evaluation, 29 ischemic stroke patients and 29 normal subjects were considered. The correctness of model depends mainly on the TP (ischemic on NCCT slices plus infarction on MR/DWI) and TN (normal on NCCT slices plus no infarction on MR/DWI).

### Performance metrics

To evaluate the performance of DL-based automatic identification model, nine performance metrics were considered including false positive (FP) rate = FP/[FP + true negative (TN)], false negative (FN) rate = FN/[FN + true positive (TP)], true negative (TN) rate = TN/[TN + False positive (FP)], sensitivity = TP/(TP + FN), specificity = TN/(TN + FP), precision = TP/(TP + FP), accuracy = (TP + TN)/(TP + FP + TN + FN), F-score = (2 × Precision × Sensitivity)/(Precision + Sensitivity), area under curve (AUC) and average precision (AP). Receiver operating characteristic (ROC) showing AUC was applied to predict the binary outcome. AP curve was plotted to represent the trade-off between sensitivity and precision (https://scikit-learn.org/stable/modules/generated/sklearn.metrics.average_precision_score.html#rcdf8f32d7f9d-1) in the case of unbalanced dataset.

## Results

### Subject recruitment

**Figure 2:**
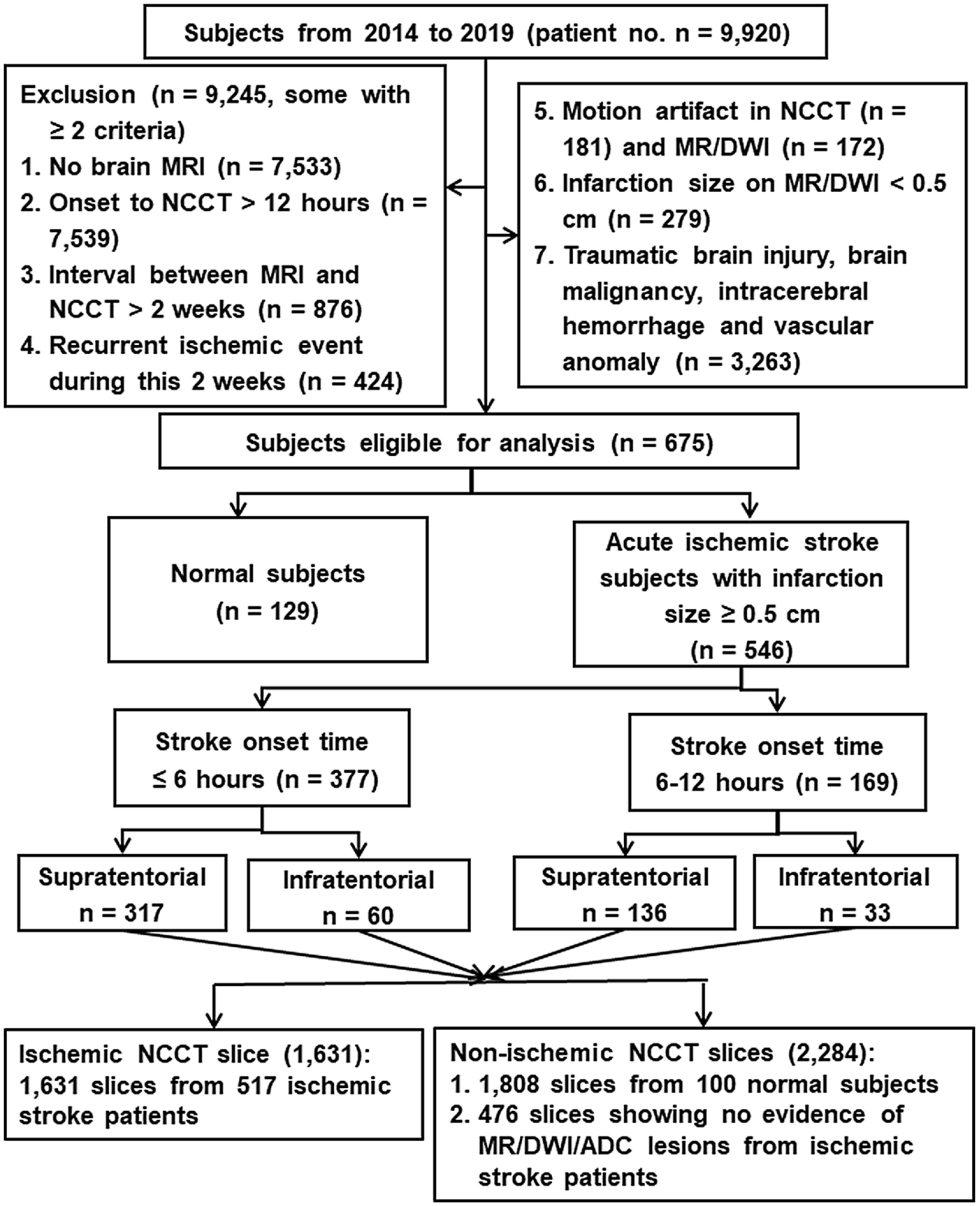
Flowchart of subject recruitment. The figure represents the inclusion and exclusion criteria for both ischemic and normal subjects enrolled along with the number of subjects and the corresponding NCCT slices considered for the present analysis from both supratentorial and infratentorial territory according to their stroke onset time. *NCCT* non-contrast computed tomogram, *MRI* MR image, *DWI* diffusion-weighted image.

Among the 9920 subjects, 546 first-ever ischemic stroke patients and 129 normal subjects (totally 675, 6.80%) met the inclusion/exclusion criteria. A total of 9245 subjects were excluded including no brain MRI in 7533, onset to NCCT time > 12 h in 7539, interval between NCCT and MR image > 2 weeks in 876, recurrent ischemic event during the 2-week interval in 424, motion artifact in NCCT in 181 and in MR/DWI in 172, infarction size < 0.5 cm on MR/DWI in 279, and traumatic brain injury, brain malignancy, intracerebral hemorrhage or vascular anomaly in 3263. In the 546 stroke patients, 377 had onset time < 6 h, and 169 between 6 and 12 h. The clinical profiles of ischemic patients and normal subjects were listed in Table 1. Ischemic lesions in different vascular territories were considered including supratentorial anterior cerebral artery, middle cerebral artery (MCA) and posterior cerebral artery territories (n = 453), and infratentorial territory (n = 93, Fig. 2).

**Table 1:** Clinical profiles of the ischemic stroke patients recruited with onset time ≤ 6 h and 6–12 h and the normal controls (NC).

	NC (n = 129)	≤ 12 h (n = 546)	P value, controls vs ≤ 12 h	≤ 6 h (n = 377)	P value, controls vs ≤ 6 h	6–12 h (n = 169)	P value, controls vs 6–12 h	P value, ≤ 6 h vs 6–12 h
Age (mean ± SD)	63.3 ± 11.2	68.2 ± 12.7	< 0.001	68.1 ± 12.5	< 0.001	68.5 ± 13.2	< 0.001	0.690
Male sex, no. (%)	77 (60.0%)	325 (59.5%)	0.972	239 (63.4%)	0.453	86 (50.9%)	0.130	0.005
Hypertension, no (%)	55 (42.6%)	435 (79.7%)	< 0.001	299 (79.3%)	< 0.001	136 (80.5%)	< 0.001	0.742
Diabetes, no. (%)	25 (19.4%)	207 (39.9%)	< 0.001	143 (37.9%)	< 0.001	64 (37.9%)	< 0.001	0.993
Hyperlipidemia, no. (%)	25 (19.4%)	238 (43.6%)	< 0.001	157 (41.6%)	< 0.001	81 (47.9%)	< 0.001	0.168
Heart disease, no. (%)	11 (8.5%)	192 (35.2%)	< 0.001	143 (37.9%)	< 0.001	49 (29.0%)	< 0.001	0.025

Statistics: Student's t test for numerical data and Chi-square test for categorical data.

### Model selection

Seven performance metrics were used to select pre-trained CNN models, and AP and AUC were used to evaluate the model performance (Table 2). Among the five pre-trained CNN models, VGG16 had the highest accuracy (0.80) and AUC (0.81). Although Inception-v3 had better AP (0.77) than VGG16 (0.75), the other performance metrics like precision (0.87), specificity (0.89), and F-score (0.78) were higher in VGG16. Based on the results of performance metrics, VGG16 was selected for further customization in model derivation phase.

**Table 2:** Performance metrics of five CNN models based on onefold data in model selection.

Architecture	Accuracy	Precision	Sensitivity	F-score	Specificity	AUC	AP
VGG16	0.80	0.87	0.71	0.78	0.89	0.81	0.75
Inception-v3	0.79	0.77	0.74	0.75	0.83	0.77	0.77
Inception-v4	0.75	0.46	0.60	0.88	0.71	0.78	0.61
IR-v2	0.74	0.92	0.63	0.74	0.91	0.78	0.60
ResNet 50	0.77	0.66	0.76	0.70	0.77	0.78	0.71

*AP* average precision, *AUC* area under curve, *CNN* convolutional neural network, *IR-v2* InceptionResNetv2, *ResNet 50* Residual Networks 50, *VGG16* visual geometry group 16.

### Model derivation

**Figure 3:**
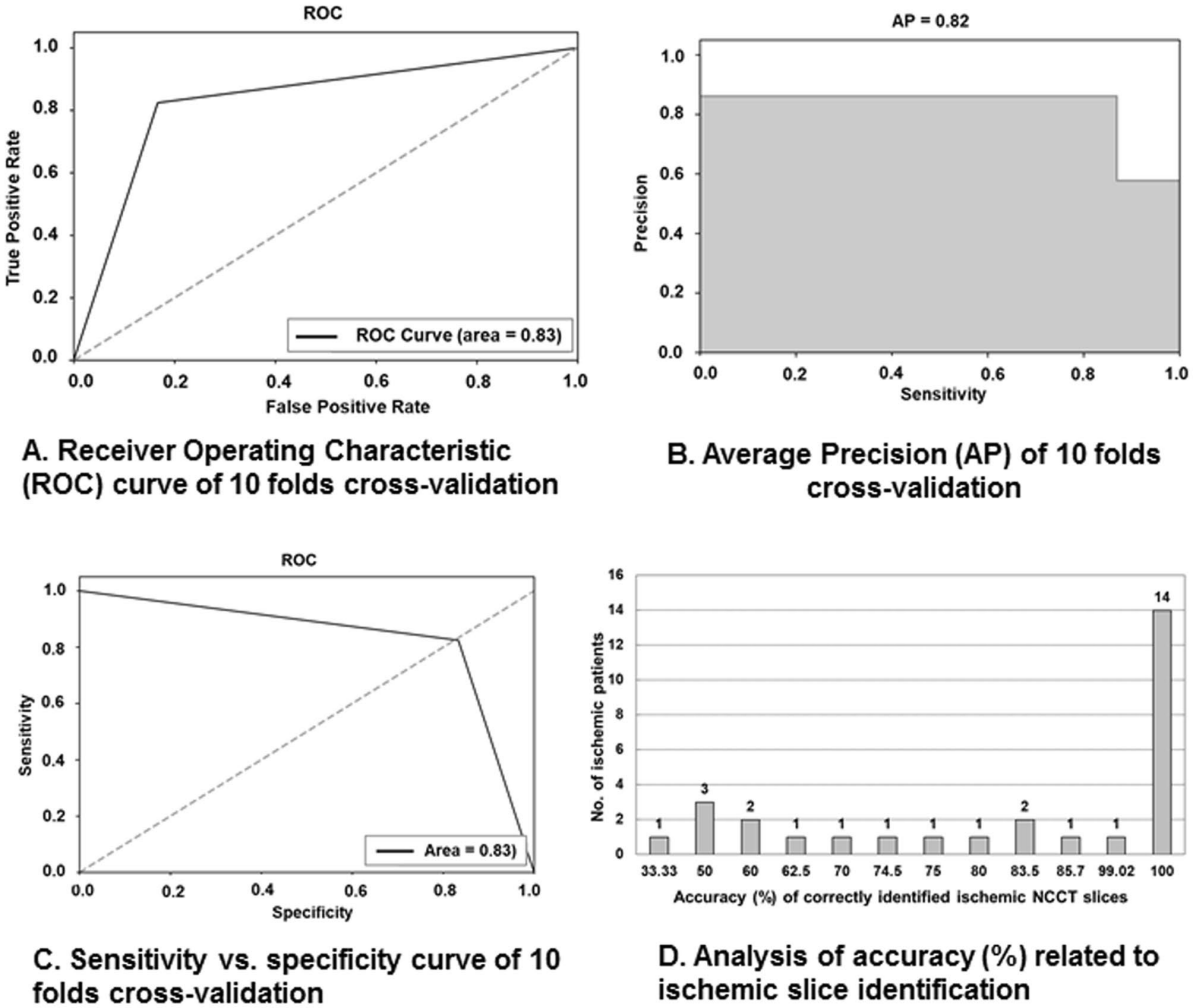
Performance metrics of customized-VGG16 convolutional neural network model. (**A**) Receiver operating characteristics (ROC) curve of tenfold cross-validation. (**B**) Average precision (AP) of tenfold cross-validation. (**C**) Sensitivity vs. specificity curve of tenfold cross-validation. (**D**) Analysis of accuracy (%) related to ischemic slice identification. *NCCT* non-contrast computed tomogram, *AP* average precision, *ROC* receiver operating characteristic, *VGG16* visual geometry group 16.

The improved results of seven performance metrics obtained after proper hyperparameter tuning of the customized-VGG16 CNN model by tenfold cross-validation process were summarized in Table 3. The average value of AUC from 10 folds was 0.83 ± 0.04. Although in Fold 2, 5 and 10, the AUC value was less than 0.8, the average sensitivity = 0.85 ± 0.08 and specificity = 0.82 ± 0.04 signified the model could recognize TP and TN accurately. In Fig. 3A, the ROC curve showing AUC = 0.83 signified the customized-VGG16 model had the ability to differentiate all positives (TP, FP) and all negatives (TN, FN). By comparing the performance metrics of the selected VGG16 model (Table 2) with the customized-VGG16 model (Table 3), the average values of F-score, sensitivity and AUC were improved from 0.78 to 0.80, 0.71 to 0.85 and 0.81 to 0.83, respectively. Except Folds 2, 5 and 10, the AUC and AP of the other folds were above 0.80 (Table 3).

**Table 3:** Performance metrics of customized-VGG16 CNN model based on tenfold data in model derivation.

Fold	Accuracy	Precision	Sensitivity	F-score	Specificity	AUC	AP
Fold 1	0.85	0.76	0.93	0.83	0.79	0.86	0.87
Fold 2	0.79	0.84	0.66	0.82	0.90	0.77	0.75
Fold 3	0.84	0.78	0.87	0.82	0.81	0.85	0.84
Fold 4	0.82	0.75	0.85	0.79	0.79	0.82	0.83
Fold 5	0.81	0.75	0.79	0.76	0.82	0.78	0.8
Fold 6	0.89	0.81	0.96	0.87	0.84	0.92	0.92
Fold 7	0.92	0.88	0.95	0.91	0.90	0.9	0.87
Fold 8	0.83	0.79	0.80	0.79	0.85	0.82	0.8
Fold 9	0.84	0.77	0.88	0.82	0.81	0.82	0.81
Fold 10	0.79	0.70	0.86	0.77	0.74	0.77	0.77
Average	0.83 ± 0.03	0.78 ± 0.04	0.85 ± 0.08	0.80 ± 0.05	0.82 ± 0.04	0.83 ± 0.04	0.82 ± 0.04

Average is expressed as mean ± standard deviation.

*AP* average precision, *AUC* area under curve, *VGG16* visual geometry group 16.

### Model evaluation

The results of customized-VGG16 CNN model for individual patient-wise analysis versus radiologists’ re-evaluation of the first-line NCCT slices were summarized in Table 4. Among the 58 subjects, 29 were confirmed to have an ischemic lesion on MR/DWI with onset time ≤ 6 h in 22 patients and within 6–12 h in 7 patients. In the case of derived CNN model, the accuracy of identifying ischemic (TP) and normal slices (TN) was 0.75 (320/425) if onset time ≤ 6 h and 0.64 (86/135) if onset time 6–12 h. In the case of radiologists, the accuracy of identifying ischemic (TP) and normal slices (TN) was 0.69 (292/425) and 0.62 (84/135) for stroke onset time ≤ 6 h and 6–12 h, respectively. It could be observed the false negative rate of identifying ischemic NCCT slices was 0.13 (30/230) in customized-VGG16 CNN model, which was lower than the visual perception of the radiologists [0.38 (87/230)]. This difference could be due to the subtle change of ischemic injury in the first-line NCCT slices which the radiologists might mistake as normal with naked eyes. Hence, some ischemic slices might be wrongly identified as normal along with the normal slices. This could justify the reason of higher true negative rate of 0.71 (233/330) and 0.62 (206/330) in the reading by radiologists and customized-VGG16 CNN model, respectively.

**Table 4:** Performance metrics of customized-VGG16 CNN model vs. radiologists for individual patient-wise analysis in model evaluation.

Patients with ischemic lesions			Customized-VGG16 CNN						Radiologists					
Stroke onset	No. patients	No. NCCT slices	TP	FP	TN	FN	Accuracy	No. correct prediction	TP	FP	TN	FN	Accuracy	No. correct prediction
≤ 6 h	22	425	161	79	159	26	0.75	22	117	63	175	70	0.69	19
6–12 h	7	135	39	45	47	4	0.64	7	26	34	58	17	0.62	6
**Subjects with normal NCCT**														
─	29	610	0	28	582	0	0.95	24	0	64	546	0	0.89	20

─, not available; *TP* true positive, *FP* false positive, *TN* true negative, *FN* false negative, *VGG16* visual geometry group 16.

Among the 29 normal subjects, the customized-VGG16 CNN model achieved the accuracy of 0.95 in identifying normal NCCT slice (582/610) with 28 normal slices being misidentified as ischemic (FP), and the false positive rate was 0.05 (28/610). In the case of radiologists, the accuracy of identifying normal NCCT slices was 0.89 (546/610) with a false positive rate of 0.10 (64/610). Although there was difference in the identification performance between the customized-VGG16 CNN model and visual perception of the radiologists, the final agreement was performed based on the subsequent MR images.

The achievement of AP = 0.82 (Fig. 3B) signified the balanced outcome of high sensitivity = 0.85 and low precision = 0.78 (Table 3). The area under curve obtained from sensitivity vs. specificity curve of tenfold cross-validation = 0.83 (Fig. 3C) representing the harmonic balance between FP and FN. Among the 29 stroke patients, the correctness of identifying ischemic lesions on NCCT slices was 100% in 14 patients and > 62.5% in 22 patients (75.9%) using the customized-VGG16 CNN model (Fig. 3D).

**Figure 4:**
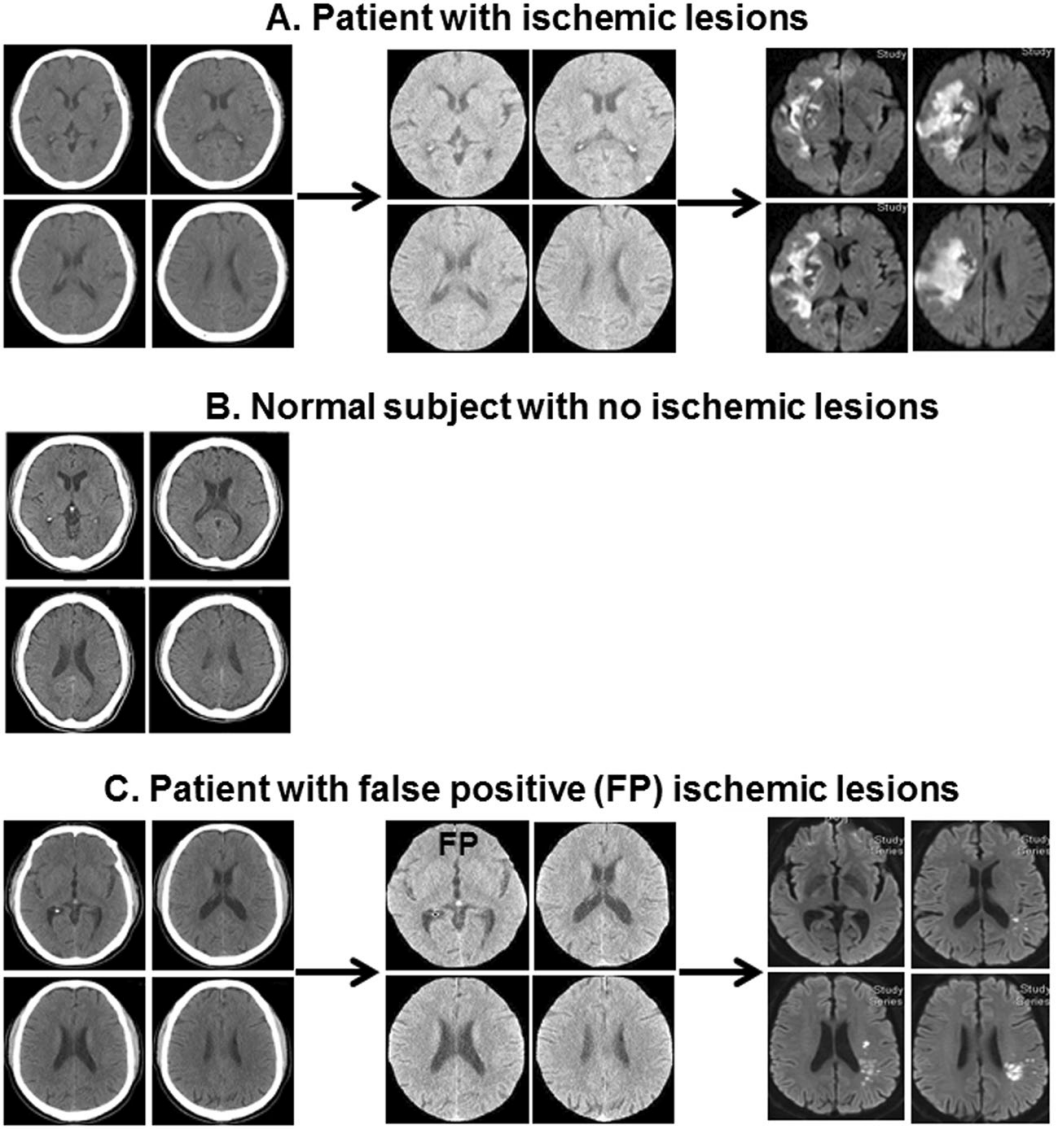
Examples of evaluation procedure identifying ischemic lesion vs. normal using derived customized-VGG16 CNN model. (**A**) Identify ischemic NCCT slices of patient with ischemic lesion and validated by corresponding MRI. (**B**) Identify the NCCT slices of normal subjects with no ischemic lesion. (**C**) Identify three correct NCCT slices of patient with ischemic lesion along with one false positive (FP), which are verified by corresponding MRI. *NCCT* non-contrast computed tomogram, *CNN* convolutional neural network, *FP* false positive, *VGG16* visual geometry group 16.

The examples of model evaluation procedure are presented in Fig. 4. The identification of ischemic from normal NCCT slices was confirmed by comparing with the corresponding MR/DWI. As shown in a stroke patient, the four ischemic NCCT slices could be identified accurately out of the entire 20 NCCT slices using the derived customized-VGG16 CNN model, which were confirmed with the corresponding MR/DWI (Fig. 4A). The model could also correctly identify the subject as normal with the absence of ischemic lesions on NCCT slices (Fig. 4B). One normal slice was wrongly identified as ischemic (FP) using the customized-VGG16 CNN model (Fig. 4C). The rest three NCCT slices were correctly recognized as ischemic (TP) which could claim the patient as an ischemic stroke patient.

## Discussion

The present study developed a supervised DL-based identification model that could correctly recognize the ischemic patients and normal subjects as well as identify the ischemic NCCT slices accurately. Our study design achieved an overall accuracy of greater than 80%, as represented in Fig. 3 and Table 3. Besides, the average values of AP = 0.82 ± 0.04 and F-score = 0.80 ± 0.05 signify the balanced outcome between lower precision = 0.78 ± 0.04 and higher sensitivity = 0.85 ± 0.08 (Table 3) even with unbalanced data.

The selection of the five CNN models in our analysis was based on the reasons including popularity, unique CNN concept, and performance. For instance, VGG16 was the 1st runner-up of the ImageNet Large Scale Visual Recognition Competition (ILSVRC) in 2014 and the winner of localization in ILSVRC 2014^19^. Also, VGG16 focused on using the filter of size 3 × 3 instead of 11 × 11 by AlexNet to reduce the number of parameters. The functional ResNet, proposed by Microsoft, was the winner of ILSVRC 2015 by introducing new approaches such as residual block, global average pooling and batch normalization^20^. The concept of ‘skip connection’ for minimization of vanishing gradient problem is also the remarkable step of ResNet. Among several ResNet variations, ResNet 50 is considered the finest CNN model for saving the computational resources and training time after ResNet18^21^. The Inception-v3 was the first runner-up of the ILSVRC 2015 challenge. The concepts of RMSProp optimizer, label smoothing, factorization and the use of auxiliary classifier are the novel additions of Inception-v3^22^. The Inception-v4 functionality is similar to Inception-v3, but the number of inception modules is higher in Inception-v4 that enables the network for deeper feature extraction^23^. InceptionResNetv2 (IR-v2) possesses the same computational complexity with Inception-v4 but can be trained much faster and achieved better accuracy with lower Top-1 error rate^23^. In our model establishment, we found customized-VGG16 CNN model could achieve the best results for the supervised DL-based identification model.

In addition, the derived model acquired the sensitivity of 86.95% in successfully recognizing 200 ischemic NCCT slices (TP) out of the total 230 slices (TP + FN, Table 4). Also, in the 29 patients with ischemic stroke (Table 4), our model could accurately recognize 25 patients having ≥ 60% ischemic slices in their NCCT series (Fig. 3D), which proved the accurate decisiveness of customized-VGG16 model in differentiating ischemic from normal slices. Although in 4 subjects, the identification accuracy of ischemic slice was ≤ 50% (Fig. 3D), the model could successfully identify at least one ischemic slice out of all NCCT series. This identification of ischemic NCCT slices from the entire pool could potentially provide valuable information in the interpretation of ischemic lesions on NCCT.

The overfitting is a major concern in any AI model performance. To avoid the overfitting issue, several precautionary methods were adopted in our derived customized-VGG16 CNN model including dropouts with dual batch normalization layers, tenfold cross-validation, re-evaluation by the radiologists and data augmentation in the form of rescaling, random rotation, horizontal flip, and zooming. As observed from Table 3, there was no large variation in the performance metrics of individual fold, implying no overfitting.

To get precise results, various convolution models were utilized. In the studies using NCCT, Chin et al. used a traditional CNN comprising of five layers to segment ischemic lesions based on CT^24^. Another study developed an automated tool by preprocessing brain CT slices using SPM8 and in-house software written in MATLAB from healthy subjects and patients, as well as statistical analyses of lesion mapping for either hemorrhagic or ischemic lesions^25^.

Previous DL-based approach on ischemic stroke used mostly brain MR image^4,6,26–35^, and some used CT perfusion^36–38^, CT angiography^39^, and ASPECTS calculation software^40^. Among the eight studies using NCCT^24,40–46^, seven studies used deep learning to detect ischemic lesions but did not mention when the NCCT was taken after stroke onset and whether infratentorial and supratentorial ischemic lesions were included for analysis^24,40–44,46^. The other one study^45^ used machine learning for detecting early infarction on NCCT which was examined within 6 h and MR image within 1 h from symptom onset. However, this study included those patients only with supratentorial MCA territory involvement. For image validation/evaluation, three studies and ours used MR/DWI scans as the reference standard^24,43,45^. One study written as a letter mentioned the use of computer-generated heat maps to indicate the possibility of infarction area^41^. A detailed comparison of these related studies^24,41–45^ including ours using the first-line NCCT is presented in Table 5.

**Table 5:** Comparison of the ischemia identification models based on NCCT.

Parameter/study	Chin et al.^24^	Gautam and Raman^42^	Jung et al.^42^	Nishio et al.^43^	Peixoto et al.^44^	Qiu et al.^47^	Pan et al.^46^	Our work
Goal	Identify ischemic lesions	Classify hemorrhagic and ischemic lesions and normal	Classify old and early ischemic lesions	Identify ischemic lesions	Classify between stroke lesions and normal	Identify ischemic lesions	Identify ischemic lesions	Identify ischemic lesions
Stroke onset time	─	─	─	─	─	< 6 h	< 9 h	< 12 h
Brain territory	─	─	MCA territory	─	─	MCA territory	Anterior and posterior territory	Whole brain
Infarction size	─	─	─	─	─	─	─	≥ 0.5 cm
Image validation	MRI	─	─	MR/DWI	─	MR/DWI	MR/DWI	MR/DWI
No. of patients	256 patch images	Hemorrhage: 18 Ischemia: 25 Normal: 31	Ischemia: 356	Suspected ischemia: 238	Hemorrhage: 100 Ischemia: 100 Normal: 100	Ischemia: 257	Ischemia: 116 Normal: 26	Ischemia: 546 Normal: 129
Accuracy	0.93	0.86	0.71	─	0.98 ± 0.02	─	0.74	0.83 ± 0.03
Sensitivity	─	─	0.62	0.373	0.97 ± 0.02	0.5	─	0.85 ± 0.08
Specificity	─	─	─	─	0.99 ± 0.01	0.98	─	0.82 ± 0.04
Precision	─	0.86	0.66	─	─	─	─	0.78 ± 0.04
F-score	─	0.86	0.71	─	0.98 ± 0.02	─	─	0.80 ± 0.05
AUC	─	─	0.73	─	─	─	─	0.83 ± 0.04
AP	─	─	0.57	─	─	─	─	0.82 ± 0.04
Limitations	Lack external validation	1. Small study population 2. Manual feature extraction 3. Visible ischemic lesion on NCCT 4. Lack external validation	Lack external validation	Lack external validation	1. Manual feature extraction 2. Visible stroke lesions on NCCT 3. Lack external validation	1. Lack external validation 2. approximation of cerebral infarction 3. Require clinical information about affected hemisphere	1. Small study population 2. Visible stroke lesion on NCCT 3. Lack of external validation	Lack external validation

─, not available; *AP* average precision, *AUC* area under curve.

Our derived customized-VGG16 CNN model for DL-based identification model is distinct and unique. First, we considered NCCT taken only within 12 h after stroke onset, since during this time period, the treatment decision for acute ischemic stroke is critical, but most ischemic lesion cannot be clearly identified on NCCT. Second, we considered not only supratentorial but also infratentorial lesions, since infratentorial ischemic lesion is especially a challenge to be identified on NCCT in the early stage of ischemic stroke. Third, we used several performance metrics with outcome visualization method and expert-level performance for model establishment.

However, there is limitation of the present study. First, all the NCCTs were collected from a single medical center, and this may raise the concerns of lack of external validation associated with the generalization of our model. Second, this study was a retrospective design, and prospective design may be needed for further validation. Third, this study only examined the presence of ischemic lesion on NCCT slice to identify the ischemic patient but did not yet examine the exact location of the ischemic lesion.

## Conclusions

The present study using CNN-based identification approach to determine the presence of ischemic lesions on NCCT demonstrated the feasibility of using deep learning in the differential identification of normal subjects and ischemic patients with stroke onset < 12 h.

## Supplementary Information

Supplementary Figures.

## Data Availability

The data used for the primary dataset, stroke code test sets and international test were obtained from hospitals as described above. Data use was approved by relevant institutional review boards. The datasets generated and/or analysed during the current study are not publicly available due to privacy issues of the patients but are available from the corresponding author on reasonable request.
