# Supplementary material for: Automatic identification of early ischemic lesions on non-contrast CT with deep learning approach
Source: Sci Rep. 2022 Oct 27;12:18054. doi: 10.1038/s41598-022-22939-x (PMC9613643; doi:10.1038/s41598-022-22939-x)
Supplement: Supplementary file 1 — Supplementary Figures. [file 41598_2022_22939_MOESM1_ESM.pdf]

Automatic Identification of Early Ischemic Lesions on Non-Contrast CT with Deep Learning Approach

Prasan Kumar Sahoo[1,3], Sulagna Mohapatra[1], Ching-Yi Wu[2], Kuo-Lun Huang[3,4], Ting-Yu Chang[3,4], Tsong-Hai Lee[3,4,*]

[1] Department of Computer Science and Information Engineering, Chang Gung University, Taoyuan, Taiwan

[2] Department of Occupational Therapy & Graduate Institute of Behavioral Sciences, College of Medicine, Chang Gung University, Taoyuan, Taiwan

[3] Department of Neurology, Linkou Chang Gung Memorial Hospital, Taoyuan, Taiwan

[4] College of Medicine, Chang Gung University, Taoyuan, Taiwan

* Corresponding author

Tsong-Hai Lee, MD, PhD

Department of Neurology, Chang Gung Memorial Hospital, Linkou Medical Center, Taoyuan, Taiwan

No. 5, Fu-Hsing Street, Guishan, Taoyuan, 333 Taiwan

TEL: 886-3-3281200, ext. 8340; FAX: 886-3-3288849;

E-mail: thlee@adm.cgmh.org.tw

Supplementary Information

Supplementary Figure S1

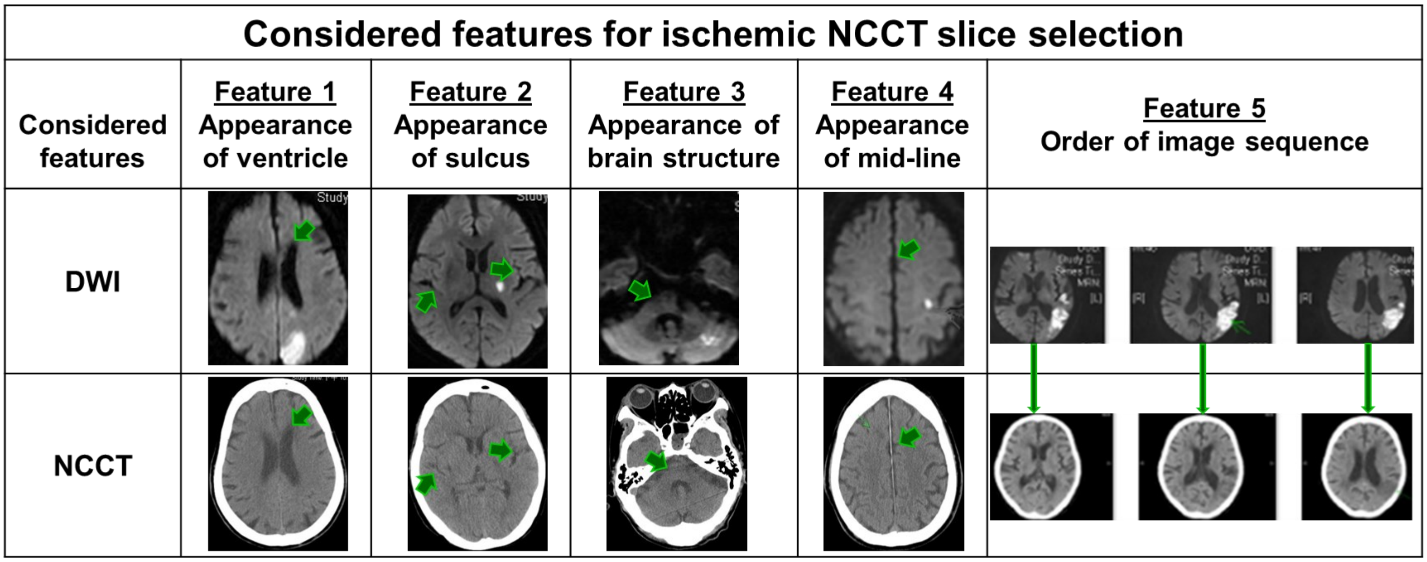

Selection of ischemic NCCT slices: Example of features for ischemic NCCT slice selection considering DWI as reference standard.

NCCT, Non-contrast computed tomogram; MRI, magnetic resonance image; DWI, diffusion-weighted image.

Supplementary Figure S2

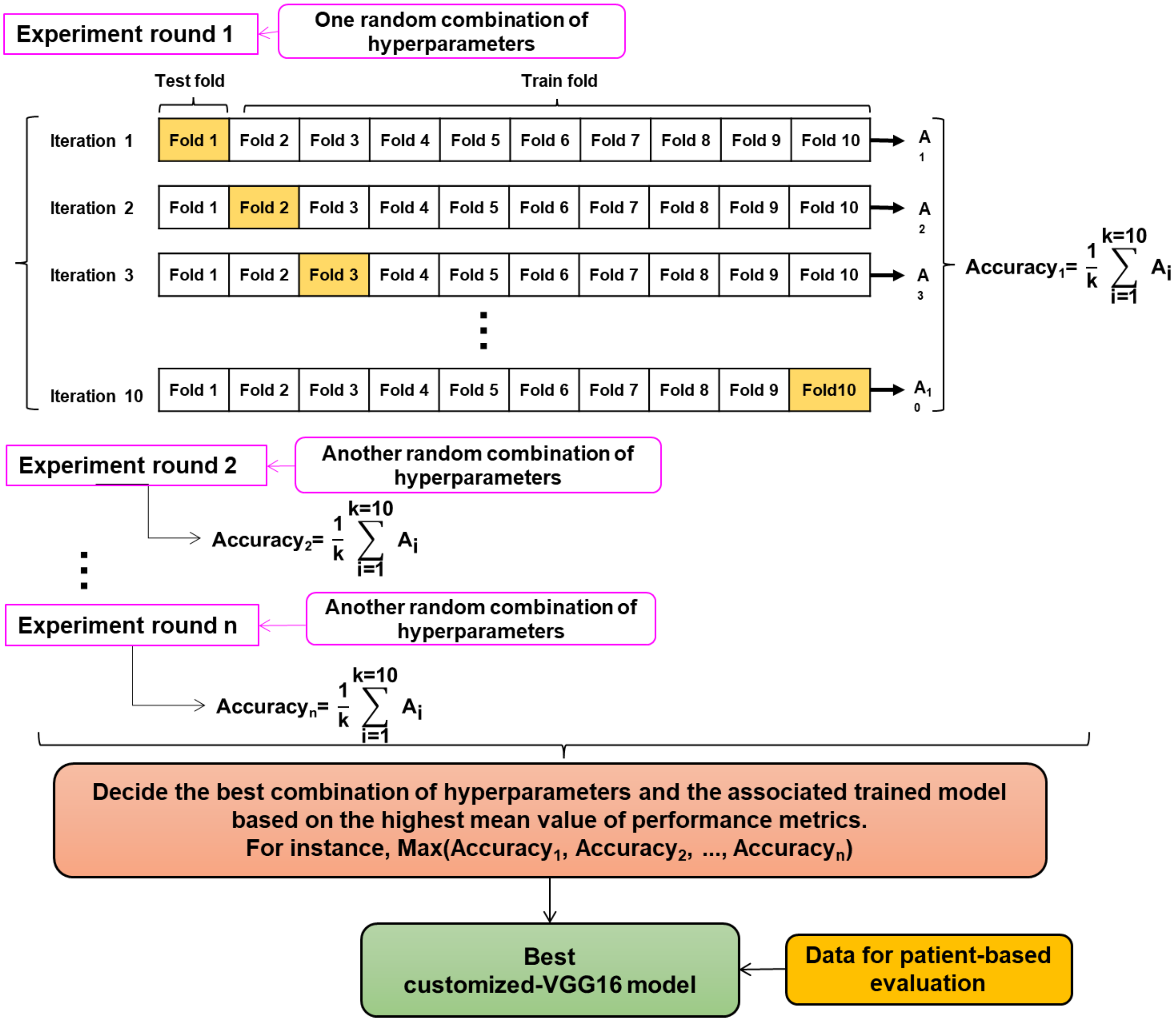

Selection of the best customized-VGG16 CNN model: An adopted strategy for deciding the best CNN (customized-VGG16) model by performing 10-fold cross-validation.

VGG16, visual geometry group 16.
